# Supplementary material for: BM-MSCs mitigate lung injury in a rat model of decompression sickness
Source: PLoS One. 2025 Aug 21;20(8):e0326618. doi: 10.1371/journal.pone.0326618 (PMC12370078; doi:10.1371/journal.pone.0326618)
Supplement: Attachment — Submitted filename: PLOS ONE COMMENTS FOR AUTHORS 3.docx [file pone.0326618.s003.docx]

**Journal**	PLOSE ONE

**Manuscript ID**	PONE-D-24-41271

**Type**	Research article

**Title**	BM-MSCs ameliorate lung injury in rat decompression sickness model**.**

**Comments and Suggestions for Authors**

The submitted manuscript explores a well-defined research topic: BM-MSCs ameliorate lung injury in rat decompression sickness model. This study represents a significant contribution to the existing body of knowledge and addresses a critical need in the field. The findings hold promise as an educational resource for understanding and managing life-threatening conditions, particularly given the rising incidence of such cases among SCUBA divers. The manuscript is well-structured, adhering to some format for original research articles and encompassing all requisite sections. While several sections are thoroughly developed, certain areas could benefit from minor revisions. Detailed comments on specific sections of the manuscript are provided below.

**Abstract**

1. Your abstract effectively highlights the key points; however, it would benefit from a more detailed explanation while remaining within the required word count.
2. Although PLOS ONE's guidelines may not explicitly address certain aspects of manuscript formatting, authors are strongly encouraged to maintain a high level of professionalism in their writing. In particular, the methodology, results, and conclusion sections should be excluded from this abstract.

**Introduction**

1. As previously noted in the abstract, line numbers are missing from the manuscript. Including line numbers is essential to facilitate reviewers in providing precise comments and suggestions for corrections. Kindly ensure that line numbers are added to the manuscript.
2. Kindly ensure that the entire manuscript is fully justified to enhance its presentation and align with professional formatting standards. This adjustment will improve readability and contribute to a polished and professional appearance.

**Methodology**

1. Subheadings should not be numbered. Authors are strongly encouraged to adhere to the prescribed guidelines to ensure their work aligns with professional standards, as this greatly enhances the credibility and presentation of their scientific contributions.

**Results**

1. The experimental setup indicates that 250 rats were used. Could the authors clarify how many mortalities were recorded during the experiment? Additionally, while the number of animals used is not excessive, it raises questions about compliance with ethical guidelines for animal research. Can the authors confirm that this quantity and the procedures used align with animal welfare regulations in China?
2. Are there alternative pro-inflammatory cytokines, chemokine genes, or pathways that could have been explored beyond those mentioned in the study? Could the authors clarify the rationale behind selecting these specific targets? Understanding the reasoning behind this choice would help address any curiosity regarding the focus of the study.

**Discussion**

1. During the model research, it was observed that mortality and incidence are strongly correlated with pressure and decompression rates. To the best of our knowledge, this is the first study to investigate the relationship between mortality and pressure rates. Could the authors specify the country or region where this study was conducted to provide better context and relevance?

References

1. The references in the manuscript are poorly formatted and do not meet the standards of typical scientific professionalism. Authors are advised to thoroughly review and restructure the references to ensure they comply with the required formatting style and reflect the expected rigor of scientific writing.
