# Supplementary material for: BM-MSCs mitigate lung injury in a rat model of decompression sickness
Source: PLoS One. 2025 Aug 21;20(8):e0326618. doi: 10.1371/journal.pone.0326618 (PMC12370078; doi:10.1371/journal.pone.0326618)
Supplement: Attachment — Submitted filename: Response to Reviewers.docx [file pone.0326618.s004.docx]

Manuscript number: PONE-D-24-56639

Ms Type: Article

Title: BM-MSCs ameliorate lung injury in rat decompression sickness model

Correspondence Author: Dr. Lin

Dear Editor:

Thank you very much for your attention and the referee’s evaluation and comments on our manuscript PONE-D-24-56639. We have revised the manuscript according to your kind advice and referee’s detailed suggestions. Enclosed please find the responses to the referees. We sincerely hope this manuscript will be finally acceptable to be published on *PLoS One*.

Thank you very much for all your help and looking forward to hearing from you soon.

Best regards.

Sincerely,

Dr. Lin

Department of Cardiology, Fuzong Clinical Medical College of Fujian Medical University, Fujian, P. R. China.

Please find the following response to the comments of referees:

Response to Reviewer #1

Thanks for your comments on our paper. We have revised our paper according to your comments:

Title and Abstract (Lines 1–20):

Line 1: The title effectively summarizes the study but could be revised for conciseness, e.g., "BM-MSCs Mitigate Lung Injury in a Rat Model of Decompression Sickness."

Response: We have revised the title to " BM-MSCs Mitigate Lung Injury in a Rat Model of Decompression Sickness ".

Line 5: Revise "is mortal disease across the worldwide" to "is a fatal disease worldwide."

Response: The phrase has been revised to "decompression sickness (DCS) is a fatal disease worldwide" for grammatical accuracy.

Line 12: The phrase "underlying mechanisms" is too broad. Specify the mechanisms targeted (e.g., oxidative stress, inflammation).

Response: We replaced "underlying mechanisms" with specific mechanisms.

Line 19: Include quantitative data (e.g., specific reductions in ROS or protein levels) to strengthen the abstract's impact.

Response: Quantitative data were added:“The ROS, CAT activities, GSH level, SOD activities and MDA ”.

Line 26: Avoid redundancy in describing decompression sickness. Consolidate phrases like "a systemic and mortal disease."

Response: Redundant descriptions were consolidated into " a systemic and mortal disease

" as your suggestion.

Line 45: The mention of mitochondrial ROS is valuable but insufficiently detailed. Explain how mitochondrial dysfunction specifically contributes to lung injury in decompression sickness.

Response: we have added “the relationship between mitochondrial dysfunction and ROS, both of which could lead to cell death and contribute to lung injury in decompression sickness” in corresponding part.

Line 72:  Provide stronger justification for using BM-MSCs by referencing prior studies demonstrating their efficacy in treating conditions involving oxidative stress and inflammation.

Response: Citations were included: "BM-MSCs’ efficacy in oxidative stress models is well-documented.

Methods (Lines 116–280):

Line 125: Clarify selection criteria for rats. Specify how the health and age of the animals ensure the validity of the model.

Response: Animal criteria clarified: " healthy *Sprague-Dawley* (SD) rats age at 8-10 weeks weighting 350 ± 10 g " in line 77.

Line 145: The decompression protocol is well-described, but explain why specific decompression rates and pressures were chosen and how they reflect clinical relevance.

Response: As you know, decompression sickness model is hard to build. The rates and pressures vary in different study. At beginning, we learned from Tang SE e tal(doi: 10.3389/fphys.2019.01616) study, during experiment we eventually found rats in DCS1 condition is suit for study in our current experiment circumstances, as in this condition DCS morbidity is guaranteed while mortality is acceptable.

Line 193: Include details of the gating strategy used for flow cytometry, as this is critical for reproducibility.

Response:

In this experiment, debris and cell clumps were excluded by FSC and SSC scatterplots, after that cell populations were delineated, and single-cell populations were selected by FSC-A/FSC-H scatterplots. Subsequently, the target cell subsets were distinguished by CD34, CD73, CD45, CD105, CD90, CD19, CD11b, HLA-DR, and CD44 markers. Finally, the expression levels of CD34, CD73, CD45, CD105, CD90, CD19, CD11b, HLA-DR and CD44 were analyzed. All gating strategies were based on unstained samples and isotype controls with negative gates and fluorescence compensation corrections.

Line 263: Justify the use of the Kolmogorov-Smirnov test for normality and describe how statistical assumptions were checked.

Response: Added: "Normality was confirmed via Kolmogorov-Smirnov test (p > 0.05), and homogeneity of variance via Levene’s test".

Results (Lines 281–459):

Line 309: Highlight reductions in the W/D ratio and BALF protein levels with quantitative comparisons to baseline or literature values.

Response: Added comparisons and quantitative data in line 242 to line 248.

Line 350: The claim that BM-MSCs "ameliorate DCS-induced lung injury" requires more specific quantitative data for support.

Response: Added comparisons and quantitative data in line 242 to line 248.

Line 392: While reductions in ROS are significant, discuss whether these changes are sufficient to prevent long-term oxidative damage or secondary complications.

Response: While ROS reduction is a cornerstone of oxidative damage prevention, its efficacy depends on addressing pre-existing damage, inflammatory cascades, and tissue-specific ROS dynamics. Long-term success requires multi-modal strategies that integrate ROS scavenging, inflammation control, and cellular repair mechanisms. Long term studies are warranted. We added in corresponding part.

Line 485: Expand the discussion on the novelty of targeting multiple cell death pathways (e.g., necroptosis, apoptosis) to highlight the study's contribution.

Response: Highlighted novelty: "First study targeting dule cell death pathways in DCS, offering a novel therapeutic paradigm".

Line 520: Explain discrepancies in the expression levels of RIPK3 and MLKL relative to lung injury severity.

Response: The discrepancies in RIPK3 and MLKL expression levels in lung injury severity arise from their distinct roles in necroptosis signaling. RIPK3's dual functionality: RIPK3 acts both as a kinase (phosphorylating MLKL to execute necroptosis) and a scaffold protein (driving inflammation independently of MLKL). MLKL as a terminal executor: MLKL phosphorylation is required for necroptotic membrane rupture, but its expression levels do not always reflect activity. This explains why RIPK3 levels may not always correlate with MLKL activation or cell death.

Line 583: Temper conclusions about BM-MSCs' "preventive potential" by addressing limitations, such as the lack of therapeutic data for post-DCS onset.

Response: conclusion added: "Prophylactic use limits clinical translation; therapeutic trials are needed post-DCS onset".

Figures and Tables:

Figure 1 (Line 288): Add overlays showing isotype controls to improve interpretability of flow cytometry results.

Response: we have incorporated isotype control overlays in Fig.1 to provide a clear visual reference for background signal discrimination.

Table 2 (Line 347): Include effect sizes and confidence intervals to enhance statistical transparency.

Response: Added effect sizes and confidence intervals to enhance statistical transparency.

Figure 6 (Line 432): Add a schematic summarizing the proposed mechanisms of BM-MSC action (e.g., antioxidant, anti-inflammatory effects).

Response: A schematic summarizing BM-MSC mechanisms was added as Fig. 7.

Language and Style:

Response: we have replaced "burst of ROS" with "a rapid increase in reactive oxygen species (ROS)."

Response: we have revised "it arouses researchers' attention" to "it has garnered significant attention."

Reviewer #2:

Thank you for your thoughtful feedback and constructive suggestions. We appreciate your recognition of our study’s significance and have carefully addressed each of your comments. Below is our point-by-point response:

1. Your abstract effectively highlights the key points; however, it would benefit from a more detailed explanation while remaining within the required word count.

Response: We have revised the abstract to provide a more comprehensive summary of the study’s objectives, key findings, and implications while adhering to the word limit. Specific details on the therapeutic mechanisms of BM-MSCs were added to enhance clarity.

2. Although PLOS ONE's guidelines may not explicitly address certain aspects of manuscript formatting, authors are strongly encouraged to maintain a high level of professionalism in their writing. In particular, the methodology, results, and conclusion sections should be excluded from this abstract.

Response: As advised, we have removed explicit subheadings (Methods, Results, Conclusion) and restructured the abstract into a unified narrative paragraph, ensuring compliance with PLOS ONE’s formatting guidelines.

1. As previously noted in the abstract, line numbers are missing from the manuscript. Including line numbers is essential to facilitate reviewers in providing precise comments and suggestions for corrections. Kindly ensure that line numbers are added to the manuscript.

Response: Line numbers have been added throughout the revised manuscript to facilitate precise referencing.

2. Kindly ensure that the entire manuscript is fully justified to enhance its presentation and align with professional formatting standards. This adjustment will improve readability and contribute to a polished and professional appearance.

Response: The text has been reformatted to full justification, improving visual consistency and alignment with professional standards.

Methodology
1. Subheadings should not be numbered. Authors are strongly encouraged to adhere to the prescribed guidelines to ensure their work aligns with professional standards, as this greatly enhances the credibility and presentation of their scientific contributions.

Response：We have removed all numbering from subheadings to align with journal guidelines.

Results
1. The experimental setup indicates that 250 rats were used. Could the authors clarify how many mortalities were recorded during the experiment? Additionally, while the number of animals used is not excessive, it raises questions about compliance with ethical guidelines for animal research. Can the authors confirm that this quantity and the procedures used align with animal welfare regulations in China?

Response: Of the 250 rats, 118 mortalities (46.83%) occurred during decompression. All procedures strictly followed China’s Animal Welfare Guidelines (GB/T 35892-2018) and were approved by the Animal Care and Use Committee of the 900th Hospital of Joint Logistics Support Force.

2. Are there alternative pro-inflammatory cytokines, chemokine genes, or pathways that could have been explored beyond those mentioned in the study? Could the authors clarify the rationale behind selecting these specific targets? Understanding the reasoning behind this choice would help address any curiosity regarding the focus of the study.

Response: The study’s focus on ROS, TNF-α, IL-1β and IL-6 aligns with their well-established roles in decompression-induced lung injury. However, additional pathways and mediators could further elucidate the mechanistic interplay. IL-8 (CXCL8): A potent neutrophil chemoattractant linked to acute lung injury (ALI) pathogenesis. IL-8 drives neutrophil infiltration and exacerbates oxidative stress, synergizing with TNF-α to amplify inflammation (PMID: 23782136). IL-10: While primarily anti-inflammatory, its suppression in decompression injury (as seen in SAP models) exacerbates inflammation. Monitoring IL-10 could highlight BM-MSC-mediated immune modulation­ (PMID: 26494447). TNF-α, IL-1β, IL-6: Core mediators of the "cytokine triad" in ALI, directly driving endothelial permeability and neutrophil activation (PMID: 37019161). NLRP3: Central to inflammasome activation, bridging oxidative stress (ROS) and IL-1β maturation (PMID: 26576075).

Discussion
1. During the model research, it was observed that mortality and incidence are strongly correlated with pressure and decompression rates. To the best of our knowledge, this is the first study to investigate the relationship between mortality and pressure rates. Could the authors specify the country or region where this study was conducted to provide better context and relevance?

Response: This study was conducted in Fuzhou, China, under controlled hyperbaric chamber (Hongyuan Oxygen Industry Co., Ltd, Yantai, China) simulating diving depths of 60-70 meters. Regional relevance is noted given China’s high prevalence of diving-related injuries.

References
1. The references in the manuscript are poorly formatted and do not meet the standards of typical scientific professionalism. Authors are advised to thoroughly review and restructure the references to ensure they comply with the required formatting style and reflect the expected rigor of scientific writing.

Response: All references have been reformatted to comply with PLOS ONE’s Vancouver style, including DOI/PMID numbers and consistent punctuation.

Reviewer #3:

Thank you for your meticulous review and valuable suggestions to improve our manuscript. Below are our point-by-point responses to your comments:
Comments:

Abstract:
1. Expand W/D (appearing for the first time)

Response: We have clarified "W/D" as "wet/dry weight ratio" upon its first mention to enhance readability.

Methods
2.  DCS model: "the rats in DCS+BM-MSCs 1d group were treated with BM-MSCs (2×10^6^cells, 200μL, saline as vehicle, tail vein injection) 1 hour (h) before DCS modeling". Is it 1 hour (h) before or 1 day before?

Response: This was a typographical error. BM-MSCs were administered 1 hour (h) before DCS modeling. The text has been corrected.

3. 2.8 Assay of oxidative stress and inflammatory markers:  "the tissue homogenate was used to detect the myeloperoxidase (MPO), malondialdehyde (MDA), superoxide dismutase(SOD), glutathione (GSH), catalase (CAT) activities by commercial reagent kits". Did the authors estimate the amount of GSH or the enzyme activity? If enzyme, please mention the enzyme name.

Response: We measured total glutathione (GSH) content using a colorimetric assay kit (Jiancheng Bioengineering Institute, Nanjing, China), not enzyme activity. This has been specified in the revised Methods section.

Results:
4. 3.1 Identification of BM-MSCs: "After osteogenic induction, alizarin red staining showed that calcium-rich extracellular matrix in the cells; after chondrogenic induction, cells were stained with Alcian blue; after adipogenic induction, oil Red O staining showed that the red lipid droplets were distributed in and between the cells (Fig. 1C-F)" Please explain the result observed with Alcian blue staining!

Response: Alcian blue staining (Fig. 1C) confirmed sulfated glycosaminoglycan (sGAG) deposition in the extracellular matrix, indicating successful chondrogenic differentiation of BM-MSCs.

5. No description about Fig. 1B in the results?

Response: Fig. 1B (now labeled Fig. 1C) shows phase-contrast microscopy images of BM-MSCs at passage 3, demonstrating their spindle-shaped morphology. Scale bars have been added.

6.  "To explore the suitable condition for the DCS rat model, we distributed rats into three groups (Fig. 2A)" Section no. is missing. If correct it should be3.2

Response: The correct section number (3.2) has been added to the figure legend.

7. The survival analysis showed that DCS significantly reduced the median survival time, but pretreatment with BM-MSCs reversed it (Fig. 2E). In addition, BM-MSCs could decrease the incidence of DCS (Fig. 2F). Please reverse the figure number in the text or reverse the figures accordingly.

Response: Figure labels 2E and 2F have been swapped to align with the text description.

8. 3.4 Pretreatment of BM-MSCs attenuates DCS induced lung inflammation: "We found that BM-MSCs ameliorated the increased serum levels of inflammation-related genes in DCS group rats (Fig. 4D-F) "inflammation-related genes or cytokines?

Response: This was an error. We measured pro-inflammatory cytokines (IL-6, TNF-α, IL-1β) in serum, not genes. The text has been revised.

9. 3.5 Pretreatment of BM-MSCs inhibits oxidative stress in DCS rats: "Compared to the Vehicle group rats, the lung ROS production increased in DCS group rats, while BM-MSCs decreased the ROS production induced by decompression (Fig. 5A)" Include figure 5B. as (Fig. 5A-B)

Response: Figure 5 includes both ROS quantification (Fig. 5A) and representative fluorescence images (Fig. 5B), labeled as Fig. 5A-B.

10. Fig. 5. : GSH activities in different group. (E)" GSH levels in different groups. (E)?

Response: The label in Fig. 5E has been changed to "GSH Levels".

11. 3.6 The effect of BM-MSCs on different types of lung cell death in DCS rats:  "pyroptosis (RIPK3 and MLKL, Fig. 6D-E), necroptosis (caspase-1 and NLRP3, Fig. 6FG)" Reverse the process for the corresponding markers. Like necroptosis (RIPK3 and MLKL, Fig. 6D-E)

Response: We apologize for the error. The corrected text reads:

- Pyroptosis: Caspase-1 and NLRP3 (Fig. E-F).

- Necroptosis: RIPK3 and MLKL (Fig. 6C-D).

12.  3.6 The effect of BM-MSCs on different types of lung cell death in DCS rats : "In this experiment, wedetected the protein expression of the markers for apoptosis (cleaved caspase-3 and Bcl-2, Fig.6B-C), pyroptosis (RIPK3 and MLKL, Fig. 6D-E), necroptosis (caspase-1 and NLRP3, Fig. 6FG) and ferroptosis (GPX4 and ACSL4, Fig. 6H-I) in lung tissues in different groups" Describe the results of DCS+BM-MSC in the result section.

Response: Added in Results 3.6: " The results showed that decompression increased the expression of cleaved caspase-3, RIPK3, caspase-1, NLRP3, GPX4, and ACSL4, while decreased the expression of Bcl-2, indicating that decompression might induced apoptosis, pyroptosis and ferroptosis. BM-MSCs pretreatment significantly reduced apoptosis (Bcl-2↑), pyroptosis (Caspase-1↓), and ferroptosis ( GPX4↑)"

Figures: 

13. Figure 1A: What is the negative control/staining control/gating control used for gating the positive cells for the corresponding marker by flow cytometry?

Response: 1. Setting of Negative Gate

The negative gate is established based on the fluorescence intensity distribution of the isotype control, typically encompassing 95–99% of the isotype control population to account for nonspecific binding and background noise.

2. Determination of Specific Signals

Cells within the experimental group exhibiting fluorescence intensity above the threshold defined by the negative gate are identified as specific staining-positive populations. This approach minimizes false-positive interpretations by distinguishing true antigen-antibody interactions from nonspecific signals.

3. Quantitative evaluation involves two primary metrics:

- Percentage of Positive Cells: Calculated as the proportion of cells exceeding the negative gate threshold relative to the total analyzed population.

- Mean Fluorescence Intensity (MFI): Reflects the average antigen density on positively stained cells, providing insights into expression levels.

14. Figure 1B. Please include the Scale bar for the microscopic pictures.

Response: Scale bars have been added to all microscopy images Fig1C-F.

15. Figure 3D. Please label the observed histological changes in the microscopic pictures.

Response: Arrows now indicate alveolar wall thickening, inflammatory cell infiltration, and hemorrhage in DCS group lungs.

16. Figure 5D. Please change the label GSH activities?

Response: The label has been revised to "GSH Levels".

17. Figure 6A. Change aspase-1 to Caspase-1 in the western blot

Response: The typo has been fixed.

Discission:

18.  "BM-MSCs scavenge overproduction reactive oxygen species[40], and increase the content of anti-oxidative enzyme, for example SOD [41], CAT [39], and GSH[42]," "anti-oxidant enzymes (such as SOD, CAT and GSH)" is the author referring levels of GSH or they referring GSH as enzyme?

Response: We confirm that GSH (glutathione) refers to the antioxidant molecule, not an enzyme. The text now states:" BM-MSCs scavenge overproduction reactive oxygen species[43], and increase the content of anti-oxidative material, for example SOD [44], CAT [42], and GSH to alleviate oxidative injury"
